# Dynamic characterization of intestinal metaplasia in the gastric corpus mucosa of Atp4a-deficient mice

**DOI:** 10.1042/BSR20181881

**Published:** 2020-02-24

**Authors:** Wei Liu, Liang-jun Yang, Yuan-liang Liu, Dong-sheng Yuan, Zi-ming Zhao, Qi Wang, Yan Yan, Hua-feng Pan

**Affiliations:** 1 Guangzhou University of Chinese Medicine, 232 Waihuan Dong Road, Panyu District, Guangzhou 510405, China; 2 Guangdong Province Engineering Technology Research Institute of T.C.M., 60 Hengfu Road, Yuexiu District, Guangzhou 510095, China

**Keywords:** atrophy, biomarker, dysplasia, H+, K+-ATPase, structure-function, Warburg effect

## Abstract

Parietal cells of the gastric mucosa contain a complex and extensive secretory membrane system that harbors gastric H^+^, K^+^-adenosine triphosphatase (ATPase), the enzyme primarily responsible for gastric lumen acidification. Here, we describe the characterization of mice deficient in the H^+^, K^+^-ATPase α subunit (Atp4a^−/−^) to determine the role of this protein in the biosynthesis of this membrane system and the biology of the gastric mucosa. Atp4a^−/−^ mice were produced by gene targeting. Wild-type (WT) and Atp4a^−/−^ mice, paired for age, were examined at 10, 12, 14 and 16 weeks for histopathology, and the expression of mucin 2 (MUC2), α-methylacyl-CoA racemase (AMACR), Ki-67 and p53 proteins was analyzed by immunohistochemistry. For further information, phosphoinositide 3-kinase (PI3K), phosphorylated-protein kinase B (p-AKT), mechanistic target of rapamycin (mTOR), hypoxia-inducible factor 1α (HIF-1α), lactate dehydrogenase A (LDHA) and sirtuin 6 (SIRT6) were detected by Western blotting. Compared with the WT mice, hypochlorhydric Atp4a^−/−^ mice developed parietal cell atrophy and significant antral inflammation (lymphocyte infiltration) and intestinal metaplasia (IM) with elevated MUC2 expression. Areas of dysplasia in the Atp4a^−/−^ mouse stomach showed increased AMACR and Ki-67 expression. Consistent with elevated antral proliferation, tissue isolated from Atp4a^−/−^ mice showed elevated p53 expression. Next, we examined the mechanism by which the deficiency of the H^+^, K^+^-ATPase α subunit has an effect on the gastric mucosa. We found that the expression of phosphorylated-PI3K, p-AKT, phosphorylated-mTOR, HIF-1α, LDHA and SIRT6 was significantly higher in tissue from the Atp4a^−/−^ mice compared with the WT mice (*P*<0.05). The H^+^, K^+^-ATPase α subunit is required for acid-secretory activity of parietal cells *in vivo*, the normal development and cellular homeostasis of the gastric mucosa, and attainment of the normal structure of the secretory membranes. Chronic achlorhydria and hypergastrinemia in aged Atp4a^−/−^ mice produced progressive hyperplasia and mucolytic and IM, and activated the Warburg effect via PI3K/AKT/mTOR signaling.

## Introduction

Metaplasia is the replacement of one differentiated cell type with another mature differentiated cell type that is not normally present in a specific tissue [1]. On the basis of epidemiological surveys, intestinal metaplasia (IM) in the stomach has been considered to be a possible precancerous state [2,3]. Based on available reports it appears that esophageal IM may lead to cancer in 1 in 860 (0.12%) individuals with this condition [4]. Notably, IM is also suggested to be involved in the development of gastric carcinomas [5] and progression to low- and high-grade dysplasia and culminate in gastric adenocarcinoma [6]. However, the pathogenesis underlying these observations remains unclear.

The secretion of hydrochloric acid in the stomach is dependent on H^+^, K^+^-adenosine triphosphatase (ATPase), a heterodimeric enzyme localized in the tubulovesicular and canalicular membranes of parietal cells [7]. This enzyme consists of two subunits: a 114-kDa α subunit (gene locus Atp4a) and a 35-kDa (protein moiety) β subunit (gene locus Atp4b) [8]. The α subunit contains ATP- and cation-binding sites and carries out the catalytic and transport functions of the enzyme [8]; it also contains sequences responsible for apical membrane localization [9]. Available reports suggest that the acid secretory activity of H^+^, K^+^-ATPase might be necessary for the viability and normal development of parietal cells [10], even for the differentiation of chief cells [11]. The inhibition of H^+^, K^+^-ATPase using omeprazole results in parietal cell degeneration and expansion of the pre-parietal cell compartment [10]. In humans, the loss of parietal cells is a prerequisite step in the development of intestinal-type gastric cancer [12]. Although the function of the gastric H^+^, K^+^-ATPase in acid secretion is well established, the importance of its acid secretory activity for the viability of parietal cells and for the normal development of the gastric mucosa is not well understood. Mouse models are a useful tool for the study of carcinogenesis as they can significantly enrich our understanding of the pathogenesis and molecular mechanisms underlying gastric IM.

It is well known that disordered metabolism, especially glucose metabolism, exists in various cancer cells [13], and thus, dysfunctional changes in cellular energy metabolism in gastric IM are worth investigating. The Warburg effect, also named as aerobic glycolysis, is a metabolic shift toward glycolysis and has been identified as being central to malignant transformation in a number of tumor types. This effect is characterized by the production of lactate to form an acid environment that creates a protective effect for cancer cells [14], which has therefore been recognized as one of the hallmarks of cancer [15]. Alterations in oncogene- and anti-oncogene-related signaling pathways are responsible for the Warburg effect in cancer cells [16]. The phosphoinositide 3-kinase (PI3K)/AKT/mechanistic target of rapamycin (mTOR) signaling pathway plays an essential part in inducing the Warburg effect in tumor cells [17] by up-regulating hypoxia-inducible factor 1α (HIF-1α) and then increasing lactate dehydrogenase A (LDHA) expression, thus shifting metabolism to aerobic glycolysis [18].

Atp4a^−/−^ parietal cells store large amounts of glycogen [19]. In this study, we used Atp4a^−/−^ mice to induce gastric IM, in a manner described previously [11]. The data arising from this study might represent a novel approach to investigate whether abnormal energy metabolism exists in gastric IM.

## Materials and methods

### Animals and reagents

**Figure 1:**
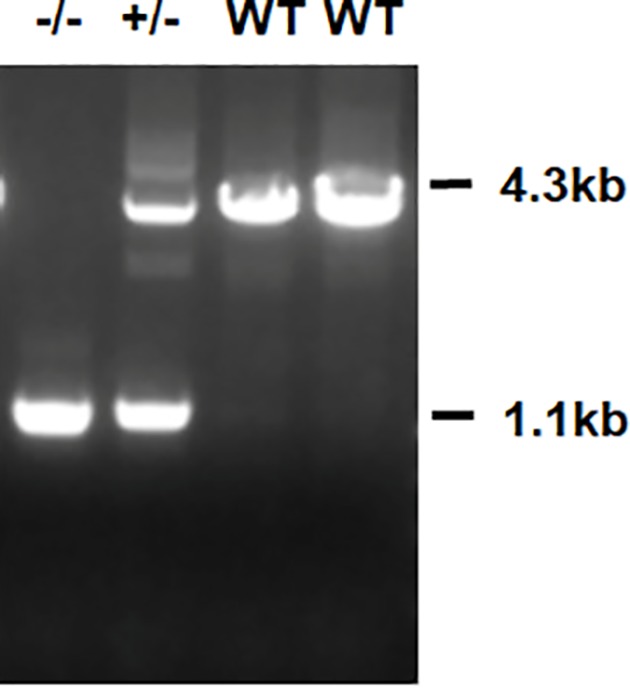
Southern blot analysis of gastric H, K-ATPase a-subunit (*Atp4a*) gene Southern blot analysis of tail DNA samples from Atp4a^−/−^ mice, heterozygous and wild-type mice.

Atp4a^–/–^ mice were produced as previously described [19]. They are devoid of a functional gastric H^+^, K^+^-ATPase enzyme, and they are achlorhydric from day 19 [19]. The Atp4a^−/−^ C57Bl/6 mice (mean weight: 20–25 g; certificate number 312024300003479) were generated by CRISPR/Cas9 (by Shanghai Model Organisms Center, Inc., Shanghai, China; No. SCXK 2014-0002). The animals were housed in a pathogen-free room under standard conditions (relative humidity: 60%, temperature: 22–25°C, 12:12-h light/dark cycle.) with free access to food and water for the duration of the study. All animal work was performed at Guangzhou University of Chinese Medicine. The experiments were performed in accordance with the National Institutes of Health Guide for the Care and Use of Laboratory Animals and were approved by The Experimental Animals Ethics Committee of the Guangzhou University of Chinese Medicine (No. S2017054). Genotypes were determined by multiplex polymerase chain reaction (PCR) with the following primers: gHKA-5′(ACAGCAGAAAGTATCTGTTGTTG), gHKA-3′(GCATAAAGGAGGGTAATGGTAG) and NEO (5′-TCCAGAATGTCCTCAATCTACT) (Figure 1).

#### Experimental model and experimental protocol

Mice were randomly divided into the following two groups: Group 1, wild-type (WT) and Group 2, Atp4a-deficient. Each group of mice was examined at the ages of 10, 12, 14 and 16 weeks without any treatment. All the experimental mice were humanely terminated with sodium pentobarbital (60 mg/kg, injected intraperitoneally), and the stomachs were removed immediately.

### Histological evaluation

The degree of the gastric mucosal lesions was judged by Hematoxylin and Eosin (H&E) histopathological examination. Tissues were dissected from selected mice, fixed in 10% formalin in phosphate-buffered saline (PBS), dehydrated, embedded in paraffin, sectioned at 5-µm and stained with H&E or with Periodic acid–Schiff (PAS) and Alcian Blue (AB) for light microscopy. The histology results were divided into four types according to the following histological features: chronic superficial gastritis, chronic atrophic gastritis, dysplasia and tumor.

### Immunohistochemistry staining

To investigate a range of important biological features, we assessed the expression of mucin 2 (MUC2) for cell differentiation, a cytoplasmic ‘neoplasm-associated’ marker (α-methylacyl-CoA racemase (AMACR)), Ki-67 for proliferation and p53 for cell cycle control. For gastric histological examination (at 10, 12, 14 and 16 weeks), stomachs were fixed overnight in 10% neutral formalin and embedded in paraffin (four to six mice per time point per genotype). An immunohistochemistry analysis was then carried out using the antibodies and detection systems described in Table 1. Immunohistochemical staining was conducted according to the protocols of the primary antibody manufacturers. Briefly, the gastral cavity was opened along the greater curvature, and carefully inspected. Then, it was cleaned with physiological saline and fixed in buffered formalin solution. A 2 cm × 1 cm tissue was resected from stomach wall in the lesser curvature near gastric antrum. Stomach sections (4 µm) were prepared using a cryostat and incubated with either mouse monoclonal antibodies or rabbit polyclonal antisera diluted in 0.05% Tween 20 with PBS for 1 h. The sections were then washed twice for 5 min in 0.05% Tween 20 with PBS and then incubated with mouse/rabbit general secondary antibody (GK5007, Dako; Copenhagen, Denmark) diluted 1:100 in 0.05% Tween 20 with PBS for 1 h. The sections were then washed as above, rinsed in water, mounted with Hematoxylin (Wexin, Guangzhou, China), and examined using a confocal microscope (Leica Microsystems DMI8 DFC7000T, U.S.A.). All immunohistochemical staining results were evaluated semi-quantitatively. Sections were classified according to the relative immunostaining as either negative (<10% of cells were positive) or positive (>10% of cells were positive) [20]. We randomly selected five high-power (400×) microscopic fields in each section for analysis.

**Table 1:** Characteristics for the antisera used for immunocytochemistry

Antigen	Dilution	Code and source
MUC2	1:100	Ab76774, Abcam
Ki-67	1:150	Ab16667, Abcam
AMACR	1:100	NBP1-87168, Novous
p53	1:100	CBL404, Millipore

Secondary antibodies with conjugates.

Mouse/rabbit general second antibody kit (GK5007, Dako; Copenhagen, Denmark).

### Western blot analysis

A total of 0.2–1 g of gastric mucosa tissue were homogenized and lysed in sample buffer (0.5 M Tris/HCl, pH 6.8, 50% glycerol, 10% sodium dodecyl sulfate (SDS), 1:100 inhibitor protease and phosphatase cocktail). The lysate was then centrifuged at 12000 rpm for 10 min at 4°C and then denatured by boiling at 100°C with 1:4 loading buffer. Equal amounts of protein from each sample (40 μg) were then separated by or 10% SDS/polyacrylamide gel electrophoresis (PAGE) and transferred to polyvinylidene fluoride membranes. The membranes were then blocked with 5% bovine serum albumin (BSA) dissolved in Tris-buffered saline with Tween 20 (TBST) for 1 h at room temperature. The membranes were then incubated overnight at 4°C with primary antibodies (anti-PI3K, Abcam: ab74136; anti-pPI3K, Abcam: ab86714; anti-phosphorylated-protein kinase B (p-AKT) (Ser^473^), Cell Signaling Technology: 4060; anti-AKT, Cell Signaling Technology: 9272S; anti-mTOR, Abcam: ab109268; anti-p-mTOR, Cell Signaling Technology: 5536; anti-HIF-1α, Millipore: MAB538; anti-sirtuin 6 [SIRT6], Norvus: NB100-2522; anti-LDHA, Abcam: ab101562). Horseradish peroxidase-coupled goat anti-mouse or anti-rabbit secondary antibody was then used for 1 h at room temperature. Routinely, protein load was monitored using a super enhanced chemical luminescence (ECL) reagent (K003, Affinity Biosciences, Cincinnati, OH, U.S.A.). Acquired images were finally analyzed using Image lab (version 3.0, Bio-Rad Laboratories, Inc, U.S.A.).

### Statistical analysis

Experimental values were presented as mean ± standard error of the mean (SEM). All statistical analyses were performed using SPSS 20.0 software (SPSS Inc., Chicago, IL, U.S.A.). One-way analysis of variance (ANOVA) was applied to analyze differences in biochemical parameters between the different groups. *P*<0.05 was considered to indicate a statistically significant difference.

## Results

### Long-term viability was not compromised by the loss of the H^+^-K^+^-ATPase α subunit gene

To evaluate the long-term effects of H^+^-K^+^-ATPase α subunit deficiency, we systemically evaluated the stomachs of Atp4a^–/–^ mice at 10, 12, 14 and 16 weeks of age compared with age-matched WT mice. These homozygous knockout mice did not show obvious developmental defects and were fertile. They also had normal behavior, weight and feeding habits. However, in the oldest Atp4a^–/–^ mice, before autopsy, the stomach was palpable and often externally visible as a protuberance in the upper left abdominal quadrant. Upon dissection, Atp4a^–/–^ mice were found to have greatly enlarged stomachs. The mucosa of the glandular stomach was pale, inflexible and clearly thicker than in WT mice, and the rugae were largely obliterated. No ulcers or papillomas were observed but the mesenteric vasculature was quite prominent. Histologically, gastric surface erosion was infrequent, but flattening of the pit region was common. Inflammation was variable and sometimes extensive. Very rarely, glandular structures were seen pressing into the submucosa.

### Spontaneous development of gastric IM in Atp4a^−/−^ mouse stomachs

WT mice showed numerous parietal cells (these cells appeared eosinophilic after H&E staining and were found in the neck region of the oxyntic glands) and zymogen cells (these cells appeared basophilic after H&E straining and were located at the gland base) with granules and regular architecture. PAS/AB staining of WT mucosa showed highly PAS-positive neutral mucins on the surface of pit cells and little, if any, AB staining.

In contrast, the gastric mucosa of the 10-week-old Atp4a^–/–^ mice was not complete, elasticity of the gastric wall was lower, and the thickness of the basement membrane was different. Within the disorganized gastric mucosal epithelial tissue, enlarged and dilated glands were discovered in the dysplastic gastric epithelial tissues. Meanwhile, gastric mucosal epithelial cells exhibited different morphological sizes and obvious heterogeneity. At the same time, the mesenchymal cells were infiltrated by inflammatory cells. Modest cyst formation was underway by 12 weeks, but with age, the formation of countless, and occasionally very large, cysts was widespread. The content of the cysts was diverse; some were empty, others were filled with various PAS-positive, PAS/AB-positive or PAS/AB-negative, hyaline-like inclusions, sloughed cells and debris. In addition to the increased number of cells per gland and cyst formation, a wide variety of hyperplastic and metaplastic cells developed in aged Atp4a^–/–^ mice. With the chronic loss of gastric H^+^, K^+^-ATPase activity, and gastric acid, mucous pit cells steadily became less PAS positive, and more AB positive, as seen by a foamy cell morphology and purple staining of the mucins at the juncture of the mucous neck and mucus pit cell zones. In Atp4a^–/–^ mice, an increase in the number of goblet-like cells was concurrent with the loss of mucous pit cell apical granules. This is generally accepted as a hallmark of IM.

At the age of 16 weeks, the glandular mucosa of Atp4a^−/−^ mice was hyperplastic, parietal cells were indistinct, and few, if any, cells with zymogen granules were present. Gastric glands were dilated and/or cystic, with various mucous and cellular contents, and various metaplastic cell types were evident, although the overall architecture was maintained for the most part. The dysplastic glands were significantly increased, irregularly arranged and weakly stained, which indicated that the abnormality of gastric epithelial dysplasia was diffusely increased in Atp4a^−/−^ mice.

**Figure 2:**
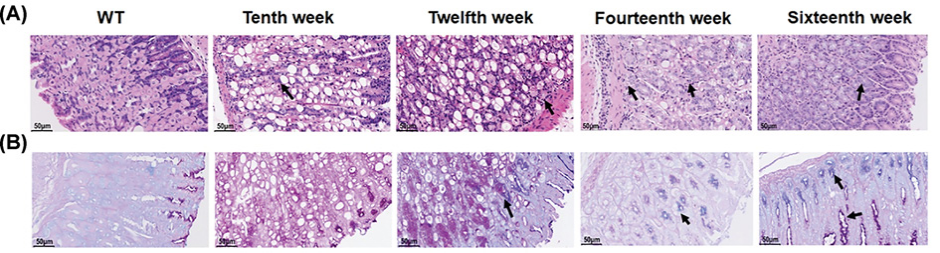
Histopathological changes of the gastric mucosa (WT; Atp4a^−/−^: Tenth, Twelfth, Fourteenth, Sixteenth week, (**A**) HE staining, (**B**) AB-PAS staining, ×400, *n*=6 mice per group).

Ciliated cell metaplasia was clearly present in 12-week-old Atp4a^−/−^ mice, and ciliated cells were most often observed toward the base of the glands that retained a relatively normal architecture, and they were located adjacent to zymogen cells. At the ages of 14 and 16 weeks, ciliated cell metaplasia persisted, and occasionally, cells that were ciliated also contained hyaline inclusions. Ciliated cells were not seen in the stomachs of the WT littermates (Figure 2).

### Characteristics of gastric precancerous lesions in Atp4a^−/−^ mouse stomachs

To determine the differences in biological features of the gastric mucosa, the abnormally localized cells in the Atp4a^−/−^ mouse stomachs were further analyzed by immunohistochemistry to study alterations in biomarkers of cell proliferation, cell differentiation and cell cycle control.

### MUC2-expressing cell lineages

**Figure 3:**
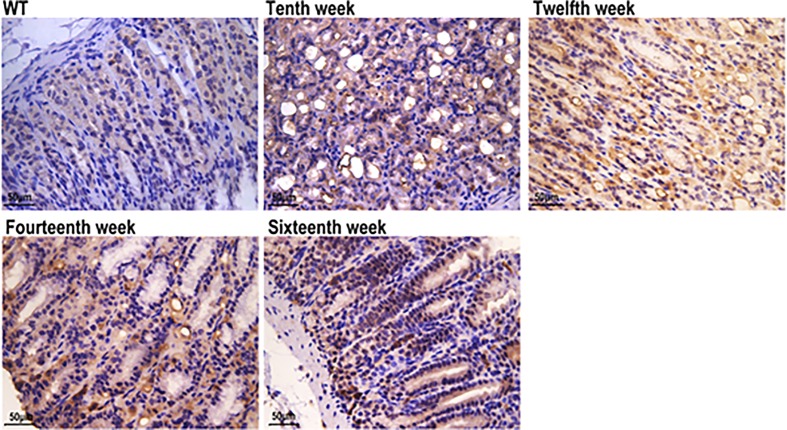
MUC2 (WT; Atp4a^−/−^: Tenth week, Twelfth week, Fourteenth week, Sixteenth week, ×400, *n*=6 mice per group)

To define mucous neck cells and centralizing metaplastic cells, a mouse monoclonal IgM antibody against MUC2 was used. Therefore, we examined tissue for MUC2 and found that the antral tissue from Atp4a^−/−^ mice exhibited strong MUC2 straining. MUC2 immunoreactivity was observed in the cytoplasm and was mainly distributed in the peripheral cytoplasm of positive cells (goblet cells) in IM. We did not observe any expression of MUC2 protein in the normal gastric mucosa of WT mice. In summary, the intensity and area of positive expression of MUC2 increased with age in Atp4a^−/−^ mice. Therefore, we concluded that in Atp4a^−/−^ mice, metaplastic tissue expresses MUC2 (Figure 3).

### AMACR- and Ki-67-expressing cell lineages

**Figure 4:**
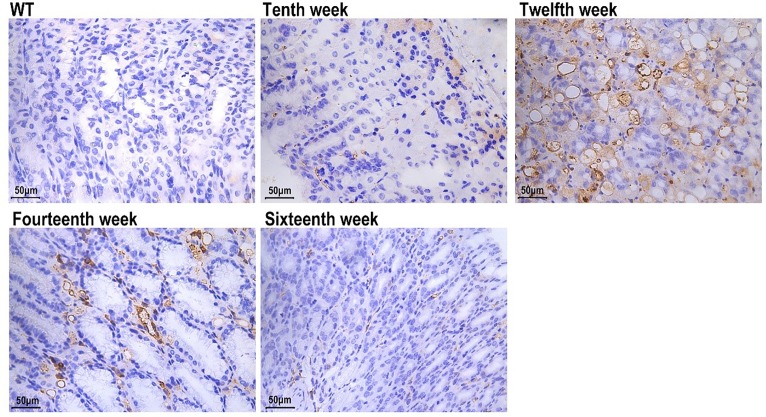
AMACR (WT; Atp4a^−/−^: Tenth, Twelfth, Fourteenth, Sixteenth week, ×400, *n*=6 mice per group)

**Figure 5:**
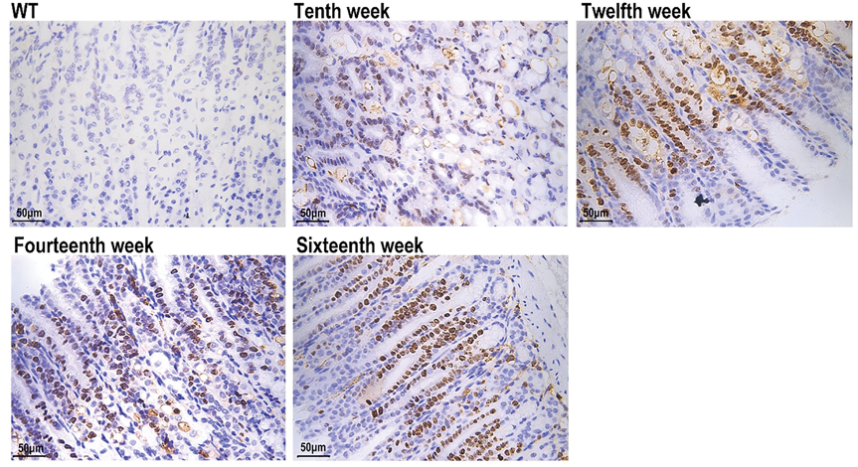
Ki-67 (WT; Atp4a^−/−^: 10th, 12th, 14th, 16th week, ×400, *n*=6 mice per group)

The AMACR staining in 12-week-old Atp4a^−/−^ mice was stronger than in other groups (Figure 4). To determine whether there were differences in proliferative and apoptotic rates between the WT and Atp4a^−/−^ mice, the expression of Ki-67 (a proliferation marker) in cells was determined. The hybridization signal with anti-Ki-67 in the gastric corpus of Atp4a^−/−^ mice was not only observed in the isthmus region (the normal proliferative zone in WT mice) but also extended along the whole neck and base of the oxyntic glands (Figure 5).

### Evidence of neoplastic transformation

**Figure 6:**
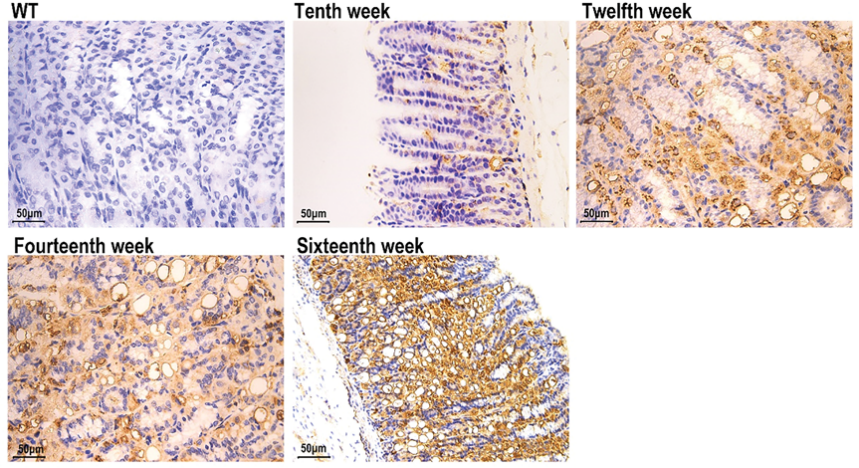
p53 (WT; Atp4a^−/−^: 10th, 12th, 14th, 16th week, ×400, *n*=6 mice per group)

Dysplastic tissue typically exhibits histological, functional and genetic evidence of transformation. Therefore, to determine whether the Atp4a^−/−^ mice exhibited such features, H&E-stained sections were examined by a pathologist. High magnification of the dysplastic areas showed loss of the normal glandular architecture, displaced cell nuclei and the presence of infiltrating mononuclear cells. To further evaluate the occurrence of malignant transformation, we examined tissues for expression of the tumor suppressor gene *p53*. The immunohistochemistry results revealed increased p53 protein expression in Atp4a^−/−^ mice (Figure 6).

### Relative expression of PI3K, p-AKT, mTOR, HIF-α, SIRT6 and LDHA based on Western blotting

**Figure 7:**
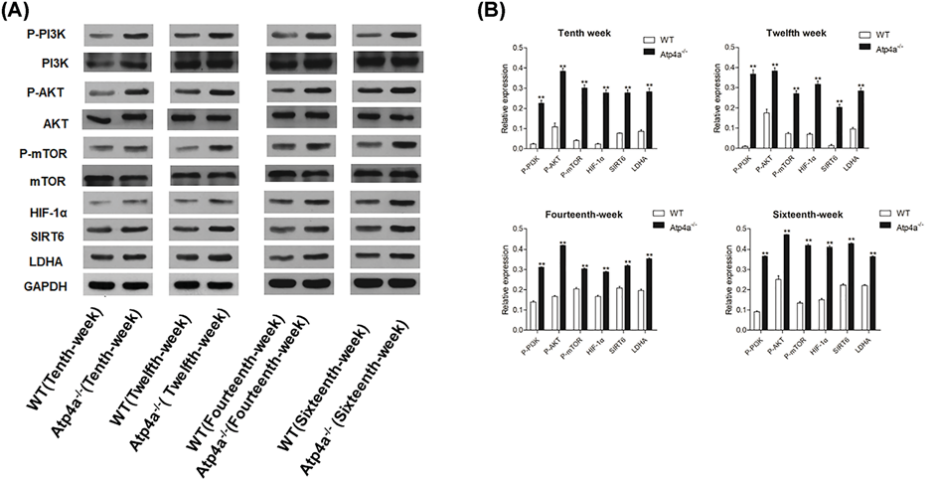
The expression of glucose metabolism-related protein in gastric mucosa in Atp4a^−/−^mice (**A**) Western blot analysis of apoptosis in the Atp4a^−/−^ mice. (**B**) The quantification of the band intensities is presented in the adjacent graphs. Data are shown as the mean ± SEM (*n*=6 mice per group). *P<0.05 and **P<0.01 versus the WT group.

Increased glucose consumption is a hallmark of cancer cells. Glucose metabolism in gastric cancer cells differs from that in normal epithelial cells. To investigate whether the Warburg effect occurred in the gastric mucosa of Atp4a^−/−^ mice, we examined the PI3K/AKT/mTOR signal pathway, which is the key regulator of the Warburg effect. The levels of these proteins were quantified by Western blot analysis. Figure 7 shows that the expression levels of PI3K, AKT, mTOR, HIF-α, SIRT6 and LDHA protein in Atp4a^−/−^ mice were significantly (*P*<0.05) increased when compared with the WT mice.

## Discussion

**Figure 8:**
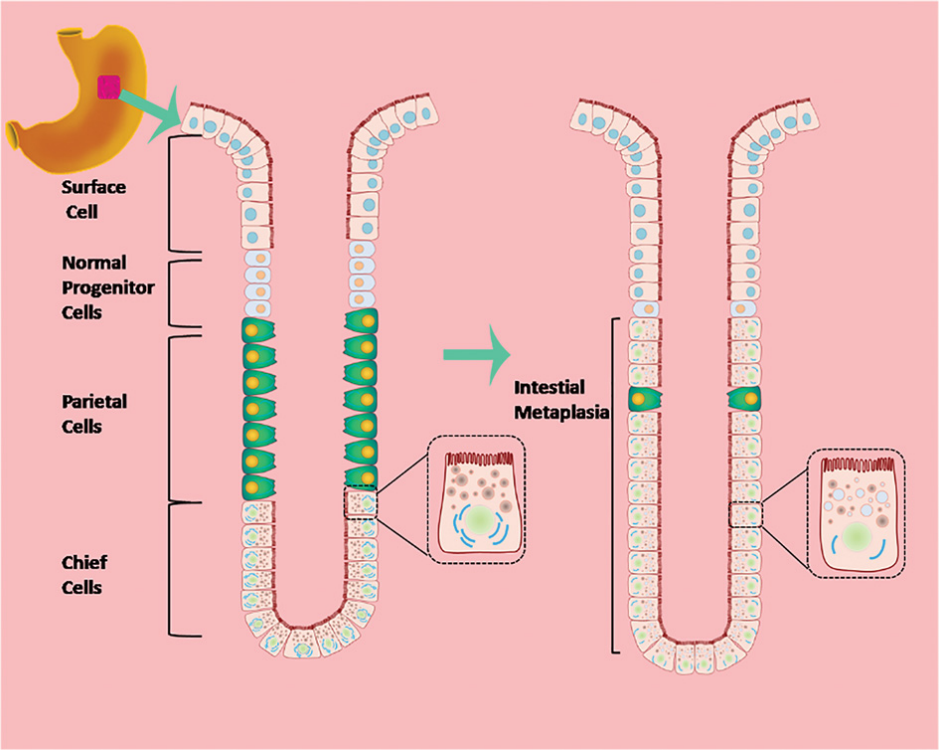
Diagram of IM of gastric mucosa

A large proportion of human gastric cancers are thought to develop from cases of mucous cell metaplasia, the prevalence of which varies with both genetic and environmental factors [20]. Critical to understanding the mechanisms involved in IM and gastric cancer, and to devising preventive and therapeutic interventions, is the need to develop an authentic animal model. Here we showed that the phenotypic progression to IM observed in the Atp4a^−/−^ mouse model is similar to the evolution of gastric cancer in humans, as initially described by Correa [21]. In both human studies and the mouse model reported here, chronic inflammation results in atrophy of the fundic mucosa, which develops into IM and dysplasia [21] (Figure 8). Intrinsic physiological differences between the two species may account for the limitations in reproducing human phenotypes in mice. Gastric IM transformation was documented by histology. In addition, the presence of IM adjacent to dysplasia was shown by the expression of MUC2, AMACR, Ki-67 and p53. Therefore, we concluded that the Atp4a^−/−^ mouse is an excellent model with which to study the development of gastric IM from atrophic gastritis. It is important that the early symptoms and the premalignant condition of the human disease are fully reproduced in our model, thus facilitating the design and evaluation of strategies for its prevention.

By the age of 12 weeks, H&E staining showed IM in the stomach of Atp4a^−/−^ mice. Atp4a^−/−^ mice are indistinguishable from their littermates at the gross level (except for the stomach of Atp4a^−/−^ mice often being externally visible as a protuberance in the upper left abdominal quadrant), and we did not encounter any premature morbidity or mortality. Thus, knockout of the H^+^, K^+^-ATPase α subunit (*Atp4a*) gene does not affect the longevity of these mice.

Although the H^+^, K^+^-ATPase α subunit is expressed in a number of organs, at this stage we only noted histopathological differences in the gastric mucosa of Atp4a^−/−^ mice. However, it is possible that the H^+^, K^+^-ATPase α subunit is essential in other organs under more strenuous physiological conditions. For example, impairment of this protein in the renal proximal tubule is known to contribute to hyperuricemia-induced renal tubular injury [22].

Variations in gastric pathology are also being described, but no consensus has been reached on how best to quantify the characteristics of metaplasia in mice, which confounds assessment in terms of cancer-predisposed states, and the relevance of these states for assessing gastric cancer risk. As the pathology is so varied, and also because the prognostic value of molecular markers for gastric IM is only just beginning to be clarified, a thorough investigation of mouse models of gastric IM has significant value. Thus, we investigated several molecular markers of gastric IM in our Atp4a^−/-^ mice.

Mucin dysregulation has also been associated with gastric cancer, and the modification of gastric mucins to those more characteristic of the intestine is associated with a poor prognosis in humans [23]. MUC2 is a colonic mucin usually expressed by goblet cells. Furthermore, MUC2 is enriched in mucinous adenocarcinoma and can be lost during the carcinogenic process in conventional adenocarcinoma. MUC2 is not expressed in normal gastric mucosa [24]. Disruption of the intestinal mucin gene, *MUC2*, in mice results in changes in crypt morphology and the development of gastrointestinal cancers [25].

AMACR, a peroxisomal and mitochondrial enzyme encoded by the *AMACR* gene, acts as a gatekeeper for the β-oxidation of dietary branched-chain fatty acids and bile acid synthesis [24]. The oncogenic role of AMACR was subsequently described in several other carcinoma types, including their precursors, albeit with variable prognostic implications [24]. AMACR has also been found to be expressed in cases of IM with dysplasia, with an incidence of 20, 40 and 80% in cases of indefinite, low-grade and high-grade dysplasia, respectively [26], but it is negative in cases of IM without dysplasia [27]. These studies suggest that AMACR represents a biomarker for precancerous lesions. Our results show the gastric of Atp4a^−/−^ mice displayed IM without dysplasia which in line with the results of H&E staining.

Ki-67 is a useful predictive and prognostic marker in cancers with a proliferation index exceeding 10–14%, delineating a high-risk prognostic category [28]. In a meta-analysis involving 5600 gastric cancer patients from 29 studies, it was concluded that high expression of Ki-67 could serve as a predictive biomarker for poor prognosis in gastric cancer patients [29].

MUC2 dysregulation was also evident in our study, indicating that acid level in the stomach may influence mucin expression. We also report the development of metaplastic and dysplastic changes and AMACR and Ki-67 dysregulation in the achlorhydric and hyperplastic stomachs of the Atp4a^−/−^ mice, independent of exposure to pathogens.

We also analyzed the expression of the p53 protein. Results showed that the p53 tumor suppressor was strongly expressed in the Atp4a^−/−^ mice compared with the WT mice. The p53 tumor suppressor acts as a sentinel for stress factors and is a regulator of crucial cellular processes, including cell cycle arrest, apoptosis, DNA repair, metabolic reprogramming, stemness, invasion and migration [30]. p53 is the most frequently mutated gene in gastric cancer (approximately 50%) [31]. It has also been shown that activation of p53 impacts glucose metabolism in cancer cells, preventing more aggressive tumor phenotypes [32]. The increase in p53 expression is accompanied by elevated Ki-67 labeling [33], which is in accordance with our study.

Cell metabolism has a central role in cell growth and survival and involves diverse signaling pathways that are regulated by intrinsic and extrinsic factors, including oncogenes, tumor suppressor genes, growth factors, pH, oxygen and nutrient levels [34]. As mentioned above, metabolic reprogramming is a hallmark of cancer, and it is closely related to other hallmarks that support malignant transformation [30]. Therefore, the role of glycolysis regulation in gastric IM needs to be further elucidated.

As previously mentioned, the microenvironment also contributes to carcinogenesis through its selective pressure, leading to adaptive advantages among the cancer cells. Evidence has indicated that hypoxic states are related to the activation and fast accumulation of HIF-1α [35]; HIF-1 inhibits mitochondrial biogenesis and favors mitophagy, thus avoiding apoptosis and enhancing therapeutic resistance [36]. HIF-1α has been proven to participate in the pathogenesis of gastric cancer through interactions with various pathways [37]. In normal cells under mild hypoxia, HIF-1 down-regulates p53 expression. Under severe hypoxia, HIF-1α activates p53, which triggers proteasome-mediated degradation of HIF-1α [38]. Overexpressed HIF-1α is a critical factor in the acceleration of malignant behaviors in gastric cancer, such as angiogenesis, invasion, metastasis and apoptosis [39]. Indeed, HIF-1α enhances glycolysis rates by up-regulating LDHA [40]. LDHA, which is a key glycolytic enzyme and catalyzes the interconversion of pyruvate and lactate, is widely overexpressed in a series of cancers including gastric cancer, and the high expression of LDHA in gastric cancer has been associated with shorter overall survival [41]. In the current study, we confirmed the expression of HIF-1α and LDHA in the Atp4a^−/−^ mice, thus suggesting that the H^+^-K^+^-ATPase α subunit promotes glycolysis by mediating the transportation of lactate.

The PI3K/AKT/mTOR pathway is currently a widely studied intracellular signaling pathway and is directly associated with cellular quiescence, proliferation, cancer and longevity [42]. Recent studies have shown that the PI3K/AKT/mTOR pathway was activated in gastric cancer and that activation of this pathway was correlated with metastasis, poor prognosis and lower survival in gastric cancer patients [43]. AKT expression directly increases the surface translocation of glucose transporters and enhances aerobic glycolysis [44]. Up-regulation of the PI3K/AKT/mTOR pathway, and increased glucose consumption via glycolysis, offer evolutionary advantages to cancer cells in normoxia as well as hypoxia. Of note, we found that the PI3K/ AKT /mTOR pathway was activated in Atp4a^−/−^ mice, indicating that glycolysis is significantly promoted by the absence of the H^+^, K^+^-ATPase α subunit. Phosphorylated AKT could inhibit the expression of p53 to promote cell proliferation and to suppress apoptosis [45]. From Western images it appears that there is not much of a difference in LDHA. Comparison between Atp4a^−/−^ mice and WT does not display differences in expression of LHDA. The fact that Akt is activated and increase in pro-proliferative molecules such as Ki67 and down-regulation of p53 indicate the proliferative nature of cells rather Warburg effect, although proliferative cancer cells show Warburg effect for ATP generation.

**Figure 9:**
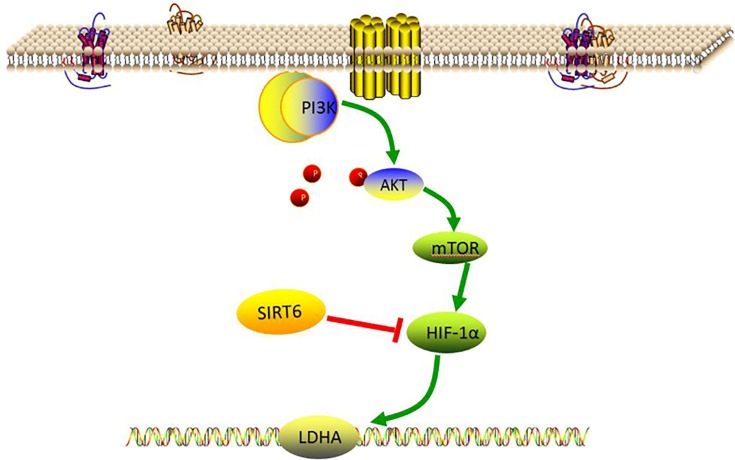
Glycolysis, relative molecules regulatory mechanism

SIRT6 plays an important role in glucose production and metabolism [46]. SIRT6 affects both gluconeogenesis and glycolysis [47,48]. Our present results showed that the expression of SIRT6 was elevated in Atp4a^−/−^ mice, which may be promoted via the PI3K/AKT/mTOR pathway to restrain the expression of HIF-α to adapt to the microenvironment of cell hypoxia (Figure 9).

Glucose metabolism in gastric cancer cells differs from that in normal epithelial cells. The up-regulation of aerobic glycolysis (the Warburg effect) in gastric cancer to meet the demands of cell proliferation is associated with genetic mutations, epigenetic modification and proteomic alteration. Metabolomic studies offer novel, convenient and practical tools in the search for new biomarkers for early detection, diagnosis, prognosis and chemosensitivity prediction of gastric IM and gastric cancer. H^+^, K^+^-ATPase is an important target for drugs that treat gastric acid-related diseases. Interfering with the process of glycolysis in cancer cells may provide a new and promising strategy for treating gastric cancer.

Our observations of parietal cells in the mucosae of H^+^, K^+^-ATPase α subunit-deficient mice of different ages show that H^+^, K^+^-ATPase is required for correct secretory membrane structure. In this, our results are consistent with the results of another study that showed that the loss of the α subunit leads to age-dependent gastric hyperplasia with incomplete IM from 3 months of age [19].

Surprisingly, the two subunits of H^+^, K^+^-ATPase have overlapping and non-overlapping functions, which have been revealed by genetic ablation studies. While α subunit-deficient and β subunit-deficient mice both develop achlorhydria and hypergastrinemia, selective loss of the β subunit also results in gastric mucosal hypertrophy and a reduced number of chief cells [11,19,49]. Our study established a connection between H^+^, K^+^-ATPase and the Warburg effect; specifically, the deficiency of the H^+^, K^+^-ATPase α subunit causes an increase in the Warburg effect. In addition, the mechanisms by which p53 promotes gastric IM involve activation of downstream PI3K/AKT/mTOR/HIF-1α signaling pathways followed by enhancement of the Warburg effect.

## Conclusion

In conclusion, the present study found that the Atp4a^–/–^ mouse appeared to provide an excellent model for studying severe glandular hyperplasia, hyaline transformation and IM, as well as the up-regulation of many factors related to cell proliferation and tumor development. We also demonstrated, unequivocally, the importance of the Warburg effect in gastric cancer development.

## Data Availability

All data generated or analyzed during the present study are included in this published article.

## Abbreviations

AB: Alcian Blue
AMACR: α-methylacyl-CoA racemase
ATPase: adenosine triphosphatase
Atp4a: H^+^, K^+^-ATPase α subunit
HIF-1α: hypoxia-inducible factor 1α
H&E: Hematoxylin and Eosin
IM: intestinal metaplasia
LDHA: lactate dehydrogenase A
MUC2: mucin 2
mTOR: mechanistic target of rapamycin
PAS: Periodic acid–Schiff
PBS: phosphate-buffered saline
PI3K: phosphoinositide 3-kinase
p-AKT: phosphorylated-protein kinase B
SDS: sodium dodecyl sulfate
SIRT6: sirtuin 6
WT: wild-type
